# Early Covid-19 vaccination rollout: a commentary from England

**DOI:** 10.1186/s13584-021-00451-3

**Published:** 2021-02-26

**Authors:** Fiona Sim

**Affiliations:** Myers JDC Brookdale Health Advisory Group, Institute for Health Research, University of Bedfordshire, Luton, UK

**Keywords:** Vaccination, Covid-19, Rollout

## Abstract

Early, rapid, nationally orchestrated vaccine rollout has been a feature in the response to the global coronavirus pandemic in Israel and the UK, two countries with long established, universal socialised health care systems. Although there are many differences between England and Israel, the factors influencing the early days of the rollout merit exploration and learning that could be of benefit to other countries as they grapple to plan their own Covid-19 vaccine programmes. This commentary considers aspects of the rollout in both countries, in response to the article by Rosen and colleagues that identified contributing and facilitating factors in Israel. Whilst vaccine procurement and authorisation has been on a UK basis, and many features of rollout have been similar throughout the UK, the details provided pertain to England.

The story of the early stages of the Covid-19 vaccine rollout in Israel makes inspiring reading. For a small country to have planned a programme, largely at the State’s expense, to vaccinate a large proportion of its population, and to have begun executing this plan with great speed during December 2020, in the midst of a substantial wave of the disease, should be celebrated.

**Table 1:** UK Priority Groups as at 30/12/20

1 Residents in a care home for older adults and their carers.	
2 All those 80 years of age and over Frontline health and social care workers.	
3 All those 75 years of age and over.	
4 All those 70 years of age and over; Clinically extremely vulnerable (CEV) individuals.	
5 All those 65 years of age and over.	
6 All individuals aged 16 years to 64 years with underlying health conditions which put them at higher risk of serious disease and mortality.	
7 All those 60 years of age and over.	
8 All those 55 years of age and over.	
9 All those 50 years of age and over.	
Source: Joint Committee on Vaccination and Immunisation https://assets.publishing.service.gov.uk/government/uploads/system/uploads/attachment_data/file/950113/jcvi-advice-on-priority-groups-for-covid-19-vaccination-30-dec-2020-revised.pdf	

Rosen and colleagues [1] have described some of the factors that they believe facilitated and contributed to the effective early rollout of the programme. All of these facilitating factors are likely to have played a part and it is as yet impossible to know precisely which are the most important features about the Israeli plan or about its population. Doubtless the assured initial vaccine supply and well organised nationwide distribution and delivery system compliant with the stringent requirements of the Pfizer vaccine were key. Logically, being geographically compact is advantageous, especially for a vaccine requiring particularly careful handling and transportation. That such a high proportion of the population had received vaccination by the end of 2020 suggests high uptake among those eligible. Although data are not provided on differential uptake, the authors refer to some differences in uptake among different societal groups, which, if not addressed, would lead to significant inequalities of population coverage. In time, with further analysis, it will become clear if the rollout achieves what we presume was the primary objective of equitable vaccine access to those most at risk of severe Covid-19.

To provide context, including a picture of the typically polarised views of Israel from the UK, in the past few weeks, Israel’s world leading vaccination programme has been greeted with mixed reactions in mainstream UK and European media, as, for example:

- a model of good practice [2]
- an example of a small country getting a complex logistical exercise right [3]
- a programme in which Israel is a guinea pig for international pharma giant, Pfizer [4]
- an example of the conduct of an unequal and oppressive regime, because residents in the autonomous Palestinian territories have not been vaccinated under the programme rolled out by the Israeli government [5]

From a UK perspective, one can make several observations. This commentary attempts to identify some areas that may be open to comparison: it makes no attempt to explore World or Middle East politics or commercial interests.

So, both Israel and the UK were quick to authorise their first vaccine against Covid-19 and to commence vaccine roll-out. Both countries had placed large advance orders for a number of potential vaccines, some of which have been authorised for use to date, with more in the pipeline. The early rollout was restricted to the Pfizer/BioNTech (‘Pfizer’) vaccine, the first vaccine to be authorised and made available in both Israel and the UK during December 2020.

In both countries, roll out plans were detailed and complex and utilised a variety of providers, among primary (family practice) and secondary (hospital) care, in the well-established socialised health care systems in both countries. Israel additionally used its national emergency care provider, Magen David Adom, best known internationally as its national ambulance service, as a source of vaccine delivery to residential facilities for older people [6], while in England, dedicated ambulance/emergency service providers were not part of the early roll out delivery plan.

Priority groups Table 1 were identified by applying evidence of risk and were similar in the two countries. So, while Israel set out explicitly how and where front line health care staff would be vaccinated (by their employers and largely at their usual places of work), in the UK, the roll out to staff has been more complicated, with hospital staff at high risk who worked in hospitals not designated as early vaccination hubs expected to seek vaccination at other venues. This led to some front line hospital staff waiting longer than others for the vaccine. This is, of course inevitable, but with prioritisation locally, it has been a cause of tension and concern.

Primary care front line staff in England were expected to be vaccinated via the local vaccination services set up through Primary Care Networks (PCNs), but individuals were subject to variability in response by those services and hospital hubs. Meanwhile, some other front line healthcare staff outside acute hospital settings (including those in mental health, ambulance services and others, such as dentists, pharmacists and optometrists), all of whom were continuing to provide daily face-to-face patient engagement, were asked to approach vaccine hubs and local services to access an appointment. In other areas, demonstrating best practice, there was an inclusive, coordinated approach led by local health systems.

At the time of writing, access to vaccine by locum (temporary clinical) primary care English National Health Service (NHS) staff and non-NHS, patient-facing healthcare professionals, remains variable. Stories made headlines occasionally when administrative personnel from health or social care organizations were found to have accessed the vaccine despite lower priority, while some other non-clinical staff, often patient-facing, such as hospital and primary care receptionists, were variably included. And, when uptake among the highest priority patient groups comprising people aged 80+ years and elderly people living in care homes, was reviewed during January, differences in geographical coverage were found. For example, London had relatively low coverage and this was unlikely to be due to poor vaccine acceptance, since uptake has been high amongst the very elderly, although concerns about ethnic differences in uptake have begun to emerge. This was relevant to policy in England, where a commitment had been made to rollout the vaccine as highest priority to care home residents and staff, following very high Covid-19 mortality among these residents in the first wave. We are not given the level of granularity in the article from Rosen and colleagues to know if similar issues occurred in Israel, or if the roll out was, indeed, as smooth as the plan itself.

With effect from 5 January 2021, the UK government announced that the standard interval between first and second doses of the two licensed vaccines, Pfizer BioNTech (‘Pfizer’) and Oxford-Astra Zeneca (‘AZ’),would be extended for everyone (with a very few clinical exceptions) from the original three or 4 weeks, respectively, as specified by the manufacturers, to 12 weeks. The rationale was that the UK was in the midst of an extensive wave of virus transmission, with rising hospital admissions and anticipated increasing death toll, emerging evidence indicating that a new variant was more contagious than its predecessor, and recognition that the NHS was close to becoming overwhelmed. On public health grounds, the decision was taken in order to vaccinate once as many people as possible in the designated priority groups, with the aim of providing at least partial and temporary immunity, and therefore reduce caseload pressure on the NHS. Thus, the second dose would be deferred by several weeks for anyone who had not received theirs by 5 January.

Although the rationale seemed plausible and was supported by many public health sources, there was outcry from various groups at this change of policy, announced without consultation -which is perhaps not surprising at the height of a very serious pandemic. Indeed, for a government whose preferred style is libertarian, this was a demonstration of uncharacteristic command and control. Firstly, came the political cynics, who believed that the policy change was all about public relations: the government being able to say that X million people had been vaccinated, where X is a lot larger in the same time period if only first doses are given. The British Medical Association, representing the country’s doctors, wanted access to the second dose both for patients who had already had their first dose and for its frontline clinician members [7]. Arguments against the longer interval were based on lack of trial evidence, with associated assertions about the efficacy of the vaccine or about its authorisation, when the second dose is delayed, and about letting down predominantly elderly patients whose second dose appointments would have to be postponed. This row still continues, with no other country, to date, adopting a similar approach to delaying the second dose. Israel, in particular, has been steadfast in its commitment to administering the second dose three weeks after the first one.

Rosen and colleagues refer briefly to problems with uptake among certain population groups, but not specifically about uptake among health and social care professionals. Perhaps one of the most concerning anecdotes emerging to date in England, and needing urgent objective analysis, is the refusal of the vaccine by substantial numbers of front line NHS health and local authority or private social care staff. The topic of mandatory vaccination has been raised in a climate of concern over vaccine hesitancy, but seems, from historical experience, very unlikely to gain any traction in UK society. In the absence of legislation, individual employers could introduce a policy to employ only those staff who had been vaccinated or who consent to vaccination, but it is too early to know the extent to which that might be pursued or the likely staff representative response. Apart from the 80+ group, uptake rates among the designated priority groups in the UK population have yet to be published, but it is among health and social care staff, including many who have witnessed the ghastly impact of this disease at close quarters, that any evidence of poor uptake must be addressed if our services are to be safer now and in future, for both users and their professional carers.

Rosen and colleagues tell us that in Israel, members of the public were invited to make their own appointments for their vaccination, using Israel’s health plans’ well-developed, secure technological capacity. In contrast, until the mass vaccination centres were activated in January 2021, in England appointments outside hospital hubs were allocated by a distinctly low-tech model, mostly by text message or mobile phone call, from Primary Care Network administration or from their family practice, to those people in the priority groups. Family practices were routinely advising other people, again by text message and reinforced via practice websites, *not* to make contact to ask about vaccination. Indeed, central guidance was issued for the public to understand the wait [8]. One wonders if behavioural experts were consulted in either country to determine if empowering and enabling people to make their own appointments, while declining those who were not from priority groups, might be more or less effective or anxiety generating than a system whose message is ‘wait to hear’ and ‘wait your turn’. For very elderly people, who might not even have a mobile phone, this wait was, anecdotally, fraught and full of uncertainty about being missed out (as indeed, a few were).

By 10 January 2021, after five weeks of roll out, geographical differences had arisen in vaccine coverage, with cover for people aged 80+ having had at least one dose reported to be 35% in England, but with London at 31% and one northern English region at 46% [9]. This pattern persisted in the following week’s data. Some differences in vaccination rates have also been observed among the four UK countries: by 23 January 2021, about 9% of the total population in England having had at least one vaccination, with less than 8% in Scotland and Wales, though some variation could be due to reporting delays. Meanwhile, the smaller proportion of elderly people as well as effective prioritisation led to Israel being able sooner than the UK to begin vaccinating people in younger and lower clinical priority groups.

Since the new year, the delivery system in England comprises the original hospital hubs and primary care services, now supplemented by mass vaccination centres and community pharmacy services. So capacity is now considerably greater and daily numbers of vaccinations had exceeded 300,000 before the end of January. There are also examples of innovative delivery mechanisms, such as a vaccine bus [10], to encourage local uptake among people who are vulnerable and difficult to reach. Within the context of a national rollout, if we are to see high rates of vaccination uptake, it seems relevant to ensure the local response is acceptable and accessible to all users, as well as evidence-based and subject to robust evaluation. So, while it makes for an untidy picture and the end result remains to be seen, it could be that, in comparison with Israel, flexibility in the model of national rollout is effective in a country like England, with larger landmass and a very much bigger, diverse population.

Another possibly influential factor in Israeli culture is that of social solidarity. Previous work in Israel on polio vaccine uptake identified social solidarity as a key feature leading to high uptake [11], whereby family members identified and engaged with the importance to their own family of each member being vaccinated. In the context of Covid-19, a campaign to promote protection of the most elderly and vulnerable among our communities might perhaps have a similar impact and could be a subject for further research.

With reference to social solidarity, culture in the UK is different. As an example, we could look at the mutual aid policy published for the NHS in January 2021 [12]. We find that only in exceptional circumstances should vaccine be transferred between providers. Providers are expected to be self-sufficient and to use all the vaccine supply they have, with sharing only if really necessary. While this may reflect good use of a scarce resource, namely vaccine, it does give the impression of a rather individualised, siloed approach to roll out and a denial of the huge amount of real-life mutual aid between workers and health care organisations that glues the NHS together at grass roots level.

## Concluding comments

Two very different countries, two rather different approaches to rapid population vaccine rollout. Both Israel and the UK are viewed as world leaders in this race to protect people by vaccination, although, as is now well known, this is only one vital component of effective pandemic response. We should encourage other countries to adopt the best of the different models and work with their own national facilitating factors to ensure many further successful national vaccination rollouts, while seeking global and just distribution of vaccine supply.

## Data Availability

N/A
